# Co-expression of B7-H3 and LAG3 represents cytotoxicity of CD4^+^ T cells in humans

**DOI:** 10.3389/fimmu.2025.1560383

**Published:** 2025-02-25

**Authors:** Yumi Tamura, Shun Ohki, Haruna Nagai, Rin Yoshizato, Shizuki Nishi, Yuqi Jin, Yasuo Kitajima, Yun Guo, Tatsuo Ichinohe, Satoshi Okada, Yohei Kawano, Tomoharu Yasuda

**Affiliations:** ^1^ Department of Immunology, Graduate School of Biomedical and Health Sciences, Hiroshima University, Hiroshima, Japan; ^2^ Department of Pediatrics, Graduate School of Medical Sciences, Kyushu University, Fukuoka, Japan; ^3^ Department of Hematology and Oncology, Research Institute for Radiation Biology and Medicine, Hiroshima University, Hiroshima, Japan; ^4^ Department of Pediatrics, Graduate School of Biomedical and Health Sciences, Hiroshima University, Hiroshima, Japan

**Keywords:** tumor immunity, CD4^+^ cytotoxic T cells, leukemia, lymphoma, EB virus, B7-H3, LAG3

## Abstract

Recent studies have highlighted the potential contribution of CD4^+^ T cells with cytotoxic activity (CD4 CTLs) to anti-tumor immunity. However, their precise roles remain elusive, partly due to the absence of specific markers defining CD4 CTLs with target-killing potential in humans. We previously demonstrated that Epstein-Barr virus (EBV)-driven immortalized B cell lines efficiently induce human CD4 CTLs with cytotoxic functions comparable to cytotoxic CD8^+^ T cells (CD8 CTLs). Here we show that EBV-driven CD4 CTLs exhibit prolonged proliferation and sustained cytotoxicity compared with CD8 CTLs, although their cytotoxic function markedly decreased during long-term culture. Comparative transcriptomic analysis of CD4 CTLs with varying cytotoxic activities identified B7-H3 and LAG3 as surface molecules associated with highly cytotoxic CD4 CTLs. Co-expression of B7-H3 and LAG3 correlated with CD107a expression and was observed on CD4^+^ T cells with enhanced cytotoxic potential in a target-dependent manner but not on CD8 CTLs. Furthermore, B7-H3^+^LAG3^+^ CD4^+^ T cells were induced during co-culture with bone marrow cells from pediatric patients with B-cell acute lymphoblastic leukemia (B-ALL). These findings suggest that B7-H3 and LAG3 co-expression represents a characteristic feature of functional CD4 CTLs in humans, providing valuable insights into the role of CD4 CTLs in tumor immunity.

## 1 Introduction

CD4^+^ T cells typically function as helper cells aiding immune responses by secreting cytokines and chemokines (1). However, recent studies have shed light on another subset of CD4^+^ T cells with cytotoxic activity (CD4 CTLs). CD4 CTLs are helper T cells that can exert direct cytotoxicity in the context of chronic viral infections or repeated antigen stimulation in the inflamed tissue or the tumor microenvironment in a major histocompatibility complex (MHC) class II-dependent manner (2, 3). Initially identified in patients with chronic viral infections, CD4 CTLs were thought to compensate for CD8^+^ T cell exhaustion (3–5). The role of CD4 CTLs in anti-tumor immunity has been underestimated due to the lack of MHC class II expression in most cell types. However, recent studies have demonstrated that certain cancer cells express MHC class II molecules in the tumor microenvironment, enabling them to be a target of CD4 CTLs (6–9). To date, CD4 CTLs have been observed in intra-tumors of various tumors, including hematological malignancies such as leukemia and lymphoma (10–14). Still, their differentiation and function in tumor control remain unclear. To better understand the role of CD4 CTLs in tumor immunity, it is important to clarify their functional state in the disease context.

EBV, a human gamma-herpesvirus that establishes lifelong latency in B cells, is known to be associated with B cell malignancies such as Hodgkin’s lymphoma and Burkitt lymphoma (15). CD4 CTLs have been implicated in monitoring the latent state of EBV in B cells, thereby suppressing the development of these malignancies (16, 17). We previously demonstrated that EBV-driven immortalized B cell lines, known as lymphoblastoid cell lines (LCLs) efficiently induce CD4 CTLs from human peripheral blood (18). In this study, we characterized the phenotypic, functional, and molecular properties of human CD4 CTLs stimulated by EBV-infected B cells, leading to the identification of surface molecules that represent their cytotoxic potential.

## 2 Materials and methods

### 2.1 Ethics statement

Experiments with samples from healthy volunteers were approved by the Institutional Review Board of Hiroshima University Graduate School of Biomedical and Medical Science. Informed consent was obtained from seven healthy EBV-seropositive donors aged between 22 and 48 years old, including two females and five males. Bone marrow aspirates from twelve pediatric patients with leukemia at diagnosis were collected. All patients provided written informed consent. The study was conducted by the declaration of Helsinki and with the approval of the Human Research Ethics Committee of Hiroshima University Graduate School of Biomedical and Medical Science. Patient characteristics are provided in the **Supplementary Materials** (**Supplementary Table 1**).

### 2.2 CD4^+^ and CD8^+^ T cells isolation from peripheral blood

Peripheral blood mononuclear cells (PBMCs) were isolated from buffy coats by density centrifugation using lymphocyte separation medium 1077 (Takara Bio). CD4^+^ and CD8^+^ T cells were either sorted using a FACSAria sorter or enriched using magnetic beads-based negative selection. For negative selection, PBMCs were washed with DMEM (Fujifilm) supplemented with 1% fetal bovine serum (FBS) and 0.1% EDTA and then stained with biotinylated CD56 (HCD56, BioLegend), CD14 (HCD14, BioLegend), CD19 (HIB19, BioLegend), CD235a (HIR2, BioLegend), CD36 (5-271, BioLegend), and either with biotinylated anti-human CD4 (RPA-T4, BioLegend) for CD8^+^ T cell enrichment or biotinylated anti-human CD8 (RPA-T8, BioLegend) for CD4^+^ T cell enrichment. After incubation for 15 minutes at 4 ℃, cells bound to biotinylated antibodies were captured using BD IMag™ Streptavidin Particles Plus (BD Biosciences) for 8 minutes, and the unbound fraction was collected. CD4^+^ and CD8^+^ T cells with at least 80% purity were used for subsequent cell culture experiments.

### 2.3 Cell culture

For the generation of cytotoxic T cells, CD4^+^ and CD8^+^ T cells separately isolated from the peripheral blood of healthy donors were cultured independently with 30 Gy-irradiated autologous lymphoblastoid cell lines at an effector to tumor ratio of 40:1 in RPMI1640 (Fujifilm) supplemented with 15% FBS (Nichirei), 1% penicillin/streptomycin (Nacalai tesque), 10 ng/ml of recombinant human IL-7 (BioLegend), and spun down at 400 rpm 2 minutes before the incubation at 37 °C. Recombinant human IL-2 (BioLegend) was added on day 4 of culture at 10 ng/ml. T cells were re-stimulated weekly with Lymphoblastoid cell lines (LCLs) at an effector-to-tumor ratio of 1:1. For patients’ samples, mononuclear cells from the bone marrow aspirates were isolated using lymphocyte separation medium 1077 (Takara Bio) and cultured in RPMI1640 supplemented with 20% FBS (Nichirei), 1% penicillin/streptomycin (Nacalai tesque), 1% non-essential amino acids (Nacalai tesque), 1% Sodium pyruvate (Nacalai tesque), 10 ng/ml of recombinant human IL-7 (BioLegend), and 10 ng/ml of recombinant human IL-2 (BioLegend).

### 2.4 Generation of lymphoblastoid cell lines

LCLs were generated as previously described (18). Briefly, PBMCs from seven healthy donors were infected with EB viral particles generated from Akata EBV (+) cells of Burkitt’s lymphoma origin, kindly provided by Dr. Hironori Yoshiyama. The obtained LCLs were cultured in RPMI1640 medium containing 15% FBS (Nichirei) and 1% penicillin/streptomycin (Nacalai tesque).

### 2.5 Killing assay

For killing assays, CD4^+^ or CD8^+^ T cells were co-cultured with autologous LCLs labelled with CellTrace™ Violet Cell Proliferation Kit (Invitrogen) at different effector to target ratios for 3.5 to 4 hours in U-bottom 96-well plates, followed by intracellular active Caspase-3 staining (BD Biosciences). The cultures were analyzed for active Caspase-3 levels in the Cell Trace-labelled target cells. The percent specific killing was calculated using the following formula: % specific killing = (% apoptotic target cells in cultures with both effectors and targets) – (% apoptotic target cells in cultures with targets alone).

### 2.6 Flow cytometry

For extracellular staining, the cells were washed with staining buffer (phosphate-buffered saline [PBS], 1% FBS, 5mM EDTA, and 0.2% NaN_3_) and stained with fluorochrome-conjugated anti-human monoclonal antibodies after blocking with Human TruStain FcX™ (BioLegend). The antibodies used for extracellular membrane staining were as follows: PE- and BV650-anti-CD4 (OKT4, BioLegend), APC/Cy7 anti-CD19 (HIB19, BioLegend), PE/Cy7 anti-CD223 (11C3C65, BioLegend), APC- and BV605-anti-CD8a (HIT8a, BioLegend), Alexa Fluor 488 anti-CD107a (H4A3, BioLegend), APC anti-B7-H3/CD276 (MIH42, BioLegend), BV650 anti-CD45RO (UCHLI, BioLegend), Alexa Fluor 700 anti-CCR7 (G043H7, BioLegend), PE anti-CD178 (FASL) (NOK-1, BioLegend), and PE anti-CD253 (TRAIL) (RIK-2, BioLegend). Intracellular staining was performed using the Cytofix/Cytoperm Fixation/Permeabilization Kit (BD Biosciences) and Zombie Aqua™ Fixable Viability Kit (BioLegend) to exclude dead cells. Antibodies for intracellular staining are as follows: BV421 anti-T-bet (4B10, BioLegend), FITC anti-Granzyme B (GB11, BioLegend), Alexa Fluor 700 anti-Perforin (B-D48, BioLegend), BV421 anti-IFN-γ (4S.B3, BioLegend). For the detection of FASL, TRAIL, and IFN-γ, CD4^+^ T cells were collected on day 21 after four rounds of restimulation with autologous LCLs, and then re-stimulated with LCLs at an effector to target ratio of 10:1. For intracellular staining with IFN-γ, CD4^+^ T cells were stimulated for 12 hours. Extracellular staining with FASL and TRAIL was performed 24 hours after the re-stimulation. All flow cytometric data were acquired using CytoFLEX S (Beckman Coulter) and analyzed using Flow Jo software (BD Biosciences).

### 2.7 T cell activation by anti-human CD3 and anti-human CD28

Each well of a 96-well flat bottom plate was coated with 2 μg/ml purified anti-human CD3 (OKT3, BioLegend) antibodies in 50 μl PBS for 2 hours at 37°C. After washing each well twice with 200 μl PBS, 1 × 10^5^ CD4^+^ T cells freshly enriched from peripheral blood were cultured in RPMI1640 medium containing 10% FBS (Nichirei) and 2 μg/ml purified anti-CD28 antibodies (clone CD28.2, BioLegend).

### 2.8 RNA sequencing

CD4^+^ T cells freshly isolated from the peripheral blood, LCL-stimulated CD4^+^ T cells with validated killing activity (from days 23-30, 4CTL-ST), and those with lost killing activity (from days 58-105, 4CTL-LT) were sorted using FACSAria II (BD Bioscience) and subjected to RNA sequencing. Total RNA was extracted using RNeasy Micro Kit (QIAGEN, Venlo, Germany) according to the manufacturer’s instructions. Libraries were prepared using BGI and sequenced using a DNBSEQ sequencer. Raw reads were subjected to quality control and adapter trimming with fastp (v0.21.0). Trimmed read mapping and quantification were performed with RSEM (v1.3.1) using STAR (v2.7.10) as an aligner. Differentially expressed genes were identified by using DESeq2 (v1.34.0). The web-based tool Metscape was used to extract candidate genes encoding membrane proteins and transmembrane proteins (19).

### 2.9 Quantitative real-time PCR

Total RNA from CD4^+^ T cells activated with anti-human CD3 and anti-human CD28 antibodies for 4 days and CD4^+^ T cells stimulated with LCLs (from days 23-30, 4CTL-ST) were isolated using ISOGEN II (NIPPON GENE) according to the manufacturer’s protocol. CD4^+^ T cells activated with 5 rounds of re-stimulation with LCLs were extracellularly stained with APC anti-B7-H3/CD276 (MIH42, BioLegend), PE/Cy7 anti-CD223 (11C3C65, BioLegend), and PE anti-CD4 (OKT4, BioLegend). Total RNA from sorted B7-H3/CD276^+^LAG3^+^CD4^+^ T cells and B7-H3/CD276^-^LAG3^-^CD4^+^ T cells were isolated using ISOGEN II (NIPPON GENE) according to the manufacturer’s protocol. Complementary DNA (cDNA) was synthesized using the ReverTra Ace qPCR Master Mix (TOYOBO) according to the manufacturer’s protocol. Real-time PCR was performed using Thunderbird SYBR qPCR mix (TOYOBO) on a Bio-Rad Real-Time PCR machine. β2-microglobulin was used as the normalization control, and data were analyzed using the ΔΔCt method. Specific primers are as follows: (B7-H3/CD276, 5’-ATGGGTGTGCATGTGGGTG-3’ and 5’-AGTGCCACCACTGGGTCT-3’ EMP1, 5’-TGGCTGGTATCTTTGTGGTCC-3’ and 5’-AAGGCCTGCACTGTCTTGAG-3’; FLT1, 5’-CGAGCCTCAGATCACTTGGTT-3’ and 5’-TGGTGGCTTTGCAGTGATAGA-3’; LAG3, 5’-TGACTGGAGACAATGGCGAC-3’ and 5’-GGGATCCAGGTGACCCAAAG-3’; SLC16A14, 5’-AATCTCTACACCCAGCAGCTC-3’ and 5’-ACCATCATCCAAGCCCATCC-3’; TSPAN13, 5’-GACACCTGTCTGGCTAGCTG-3’ and 5’-TGTAGGTCAGCCAAACACCC-3’; MCAM, 5’-CGCTACCTGTGTAGGGAGGA-3’ and 5’-GGGACGACTGAATGTGGACC-3’; CD200, 5’-GGATGCCCTTCTCTCATCTGT-3’ and 5’-CATCCTGGGTCACCACTTGC-3’; EVC, 5’-AGGCAGGAGTCATGGACCTT-3’ and 5’-GCTGAGTGAGCCTGAGGTCTG-3’; TBX21, 5’-CATTGCCGTGACTGCCTACC-3’ and 5’-GATGCTGGTGTCAACAGATGTG-3’; ZEB2, 5’-AGCCTCTGTAGATGGTCCAG-3’ and 5’- GTCACTGCGCTGAAGGTACT-3’; IFNg 5’-TTGGCTTAATTCTCTCGGAAACG-3’ and 5’-CGCTACATCTGAATGACCTGC-3’; RUNX3 5’-GGTGGCCAGGTTCAACGA-3’ and 5’-TGATGGTCAGGGTGAAACTCTTC-3’.

### 2.10 Statistical analysis

Data were analyzed with GraphPad Prism software version 10.0. The results are represented as the mean ± SD. *p* values were calculated by one-way ANOVA, two-way ANOVA or student`s t-test. Statistical significance was determined with alpha<0.05 and presented as *, <0.05; **, <0.01; ***, <0.001; ****, <0.0001. Pearson’s correlation analysis was used to calculate correlation coefficients.

## 3 Results

### 3.1 CD4 CTLs proliferate longer than CD8 CTLs but lose their activity upon repeated stimulation

**Figure 1:**
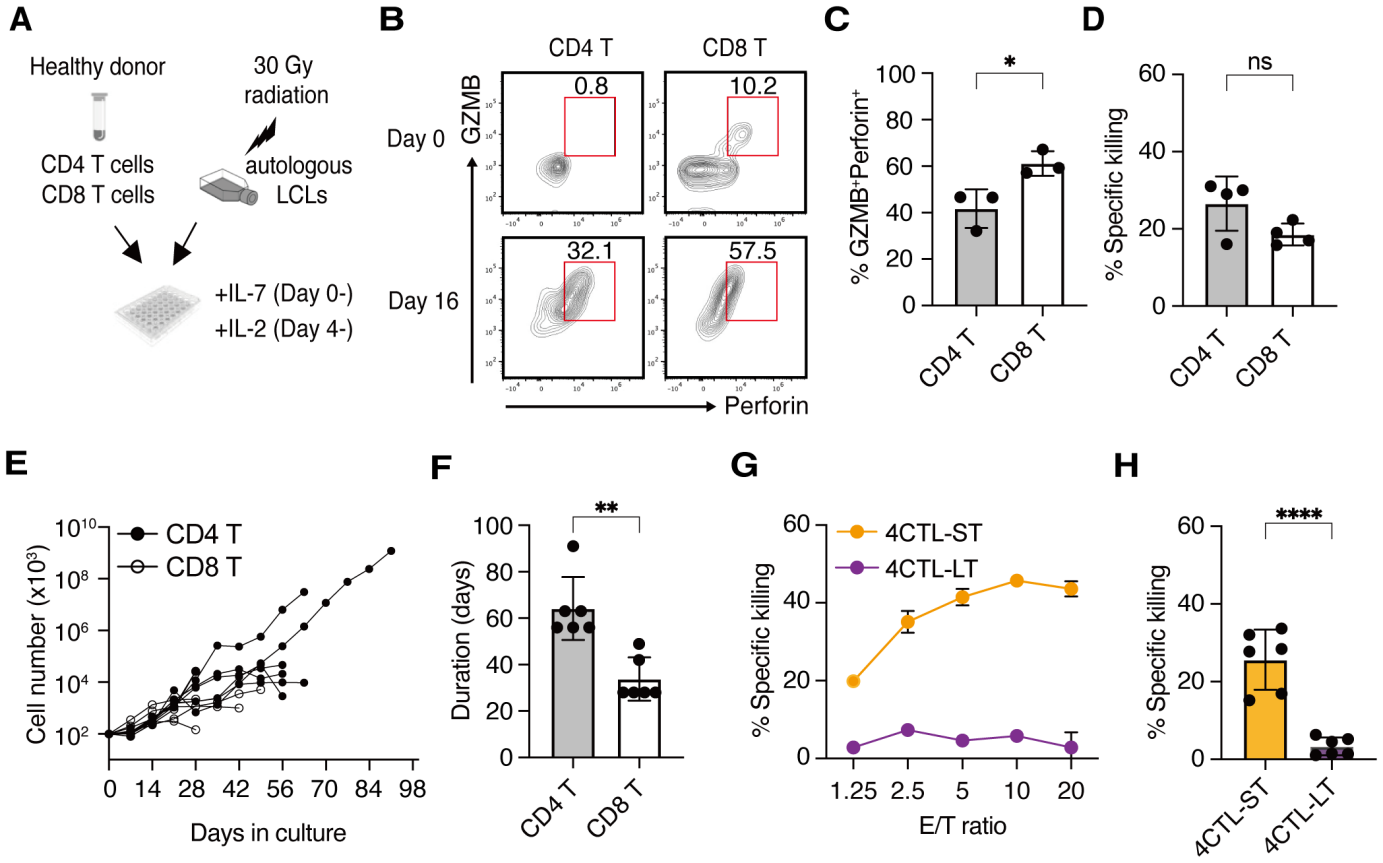
CD4 CTLs proliferate longer than CD8 CTLs but lose their activity upon repeated stimulation **(A)** Schematic representation: CD4^+^ and CD8^+^ T cells were two-way-sorted from the peripheral blood T cells from healthy donors and independently stimulated weekly with irradiated autologous LCLs generated from the same donor. IL-7 and IL-2 were added to the culture medium from day 0 and day 4, respectively. **(B)** Representative flow cytometry plots for the expression of Granzyme B (GZMB) and Perforin on day 0 (*upper panels*) and day 16 (*lower panels*) of stimulation with autologous LCLs in the CD45RO^+^CD4^+^ compartment (*left panels*) and CD45RO^+^CD8^+^ compartment (*right panels*). Percentage in the gate is shown. **(C)** Percentage of cytotoxic T cell population defined by the co-expression of GZMB and Perforin at day 16-30 of CD4^+^ and CD8^+^ T cells (n=3). **(D)** Percentage of specific killing activity at day 16-30 of CD4^+^ and CD8^+^ T cells (n=4) against autologous LCLs. Results of the 10:1 effector to target (E/T) ratio are shown. **(E)** Accumulated cell number of CD4^+^ (*filled circles*) and CD8^+^ (*open circles*) T cells from 6 independent experiments involving 4 healthy donors (n=6). **(F)** Proliferative duration of CD4^+^ and CD8^+^ T cells (n=6). **(G)** Representative cytotoxic activity of CD4^+^ T cells on day 30 (4CTL-ST) and day 68 (4CTL-LT) at the indicated E/T ratio. **(H)** Cumulative analysis of killing activity for CD4^+^ T cells from days 16-30 (4CTL-ST) and days 56-105 (4CTL-LT) from 6 independent experiments involving 4 donors (n=6). Results of the 10:1 effector to target (E/T) ratio are shown. Data are mean ± SD with statistical significance determined by unpaired t-test **(C, D, F, H)** and paired t-test **(I–K)**. The *p*-values are represented as *p < 0.05; **p < 0.01; ***p < 0.001; ****p < 0.0001.

When CD4^+^ and CD8^+^ T cells were simultaneously isolated from the peripheral blood of the same donor and were co-cultured independently with irradiated autologous LCLs by weekly restimulation (**Figure 1A**), a substantial proportion of both T cell subsets expressed Granzyme B (GZMB) and Perforin, hallmark molecules of cytotoxic T cells, by day16, indicating their differentiation into CTLs (**Figure 1B**). Similar results were observed across donors (**Figure 1C**). Although the frequency of CTLs was lower in the CD4^+^ T cell population compared to CD8^+^ T cells (**Figure 1C**), their cytotoxic activity against LCLs was comparable (**Figure 1D**). Subsequent analysis revealed that CD4 CTLs relatively outperformed CD8 CTLs in accumulated cell numbers and proliferative duration, despite variations among donors (**Figure 1E**). CD8 CTLs lost proliferative capacity in an average of 33 days, whereas CD4 CTLs exhibited an average proliferative duration exceeding 56 days, with the longest reaching up to 105 days (**Figure 1F**). These data suggest that LCL-induced CD4 CTLs exhibit prolonged proliferative capacity and delayed exhaustion relative to CD8 CTLs. In alignment with this, short-term cultured CD4 CTLs (4CTL-ST, 2–5 rounds of LCL restimulation) exhibited robust cytotoxic activity. However, long-term cultured CD4 CTLs (4CTL-LT, >8 rounds of LCL restimulation, >56 days of culture) displayed a marked reduction in cytotoxic activity, coinciding with growth arrest (**Figures 1G, H**).

### 3.2 Transcriptome analysis identifies B7-H3/CD276 and LAG3 discriminating functional CD4 CTLs

**Figure 2:**
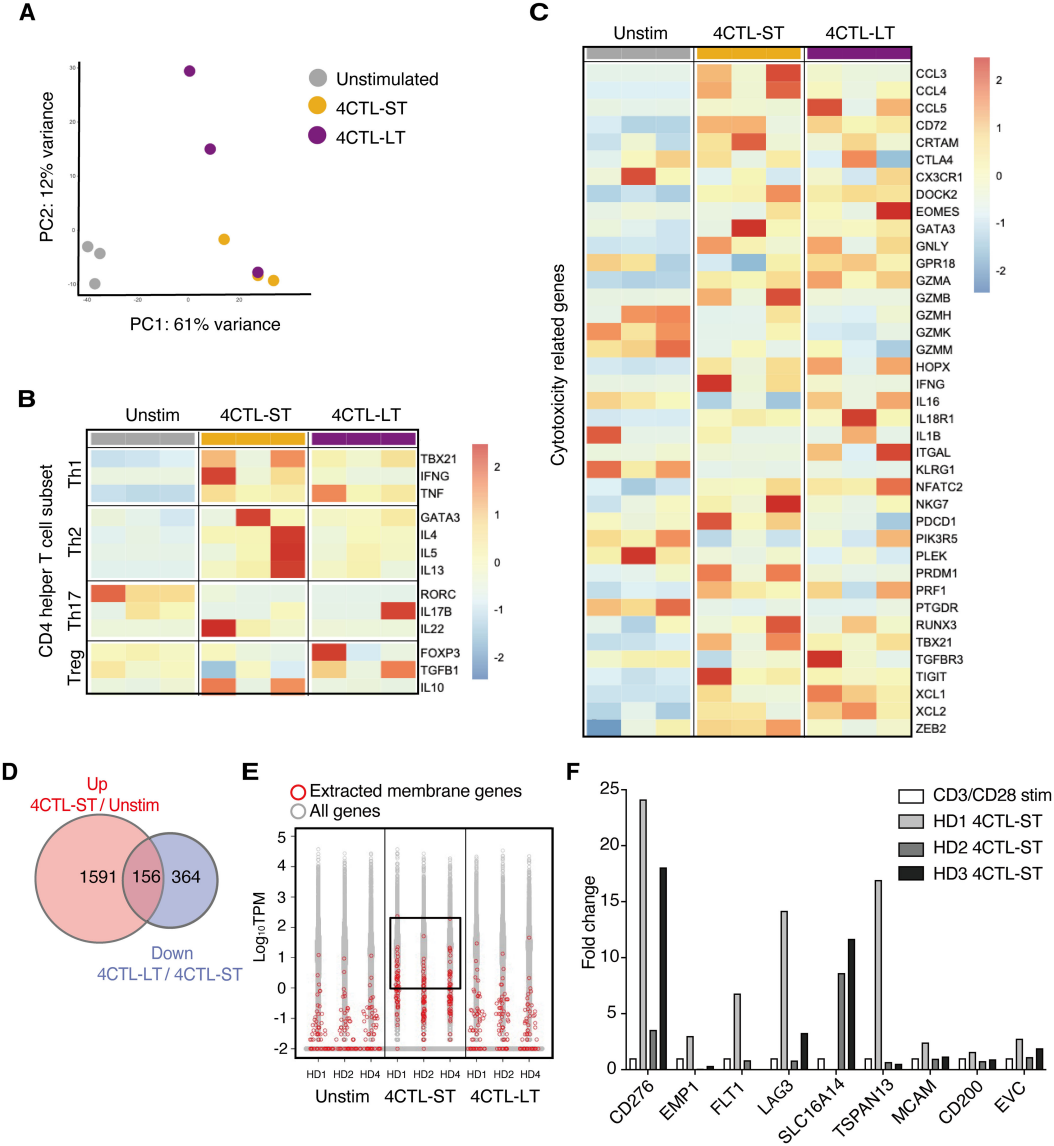
Transcriptome analysis identifies B7-H3/CD276 and LAG3 discriminating functional CD4 CTLs **(A)** PCA plots of unstimulated CD4^+^ T cells, 4CTL-ST (at days of 23-30), and 4CTL-LT (at days of 58-105) from three healthy donors. The top 10,000 genes with the highest variance were used to plot the PCA. **(B, C)** Heatmap of gene expression related to helper CD4^+^ T cell subsets **(B)** and cytotoxicity of lymphocytes **(C)** in indicated CD4^+^ T cell fractions. Color code values indicate relative expression levels shown as TPM. **(D)** Venn diagrams of DEGs upregulated in 4CTL-ST to unstimulated CD4^+^ T cells, and DEGs downregulated in 4CTL-LT to 4CTL-ST. The numbers of genes are indicated in each compartment. **(E)** The distribution of expression levels (measured as TPM) of all RNA-sequenced genes (*in gray)* and genes encoding the surface membrane proteins (*in red*) in the indicated samples. Log_10_ TPM values greater than 0 in 4CTL-ST are enclosed. **(F)** Fold change in gene expression of the indicated molecules, normalized to β2 microglobulin, in 4CTL-ST as measured by quantitative real-time PCR (n=3). Bars show relative gene expression compared to CD4^+^ T cells stimulated with CD3/CD28 for 4 days.

To investigate the transcriptional differences underlying functional variations in CD4 CTLs, we performed RNA sequencing on unstimulated CD4^+^ T cells, 4CTL-ST, and 4CTL-LT cells from three healthy donors. Principal component analysis (PCA) revealed that 4CTL-ST and 4CTL-LT clustered separately from unstimulated CD4^+^ T cells but were closely related (**Figure 2A**), suggesting similar overall transcriptional patterns between the two CTL populations. As previously reported, gene expression levels of transcription factors, cytokines, and characteristics in helper CD4^+^ T cell subsets revealed that LCL-activated CD4^+^ T cells expressed genes important for other helper T cells, such as *TBX21* (for Th1), *GATA3* (for Th2), and *FOXP3* (for Treg), although the expression patterns were highly biased among donors (**Figure 2B**) (20–22). Among these transcription factors, T-bet (encoded by *TBX21*) expression was significantly lower in 4CTL-LT at the protein level, consistent with previous reports linking it to the cytotoxic activity of CD4 CTLs (**Supplementary Figure 1A**) (23). Next, we compared the expression of cytotoxicity-related genes previously reported in CD4 CTLs. While gene expression related to cytotoxicity was significantly altered in 4CTL-ST and 4CTL-LT compared to unstimulated CD4^+^ T cells, these profiles did not differ substantially between the two CTL subsets, although *GZMB* showed a tendency to decrease in 4CTL-LT (**Figure 2C**) (2, 14). Consistent with this, intracellular staining of GZMB and Perforin showed variable expression albeit with lower expression in 4CTL-LT, compared to 4CTL-ST (**Supplementary Figures 1B, C**). Collectively, these results suggest that 4CTL-ST and 4CTL-LT are transcriptionally similar, making it challenging to discriminate functional CD4 CTLs based solely on the expression profiles of the known cytotoxicity-related genes.

Then we sought to investigate surface molecules that associate with functional CD4 CTLs with target-killing capacity using these transcriptome data. First, we extracted 156 genes that were significantly upregulated in 4CTL-ST compared to unstimulated CD4^+^ T cells and downregulated in 4CTL-LT compared to 4CTL-ST (**Figure 2D**). Of the 51 genes encoding membrane proteins, 11 genes were extracted with a cutoff of Log_10_ TPM greater than 0 in all three donors (**Figure 2E**). Finally, 9 genes encoding the cell surface membrane proteins were selected and the gene expressions were analyzed by quantitative real-time PCR (qRT-PCR), which confirmed the results obtained from the RNA-seq data (**Supplementary Figure 2**). To select genes specific to CD4 CTLs, the relative expression of these candidate genes was compared with that of CD4^+^ T cells stimulated with anti-CD3 and anti-CD28 antibodies as control (**Figure 2F**). Among the genes elevated in two or more donors, namely *CD276*, *LAG3*, and *SLC16A14*, we focused on *CD276* and *LAG3*, which are known for their immune-related function.

### 3.3 B7-H3 and LAG3 co-expression correlates with functional CD4 CTLs

**Figure 3:**
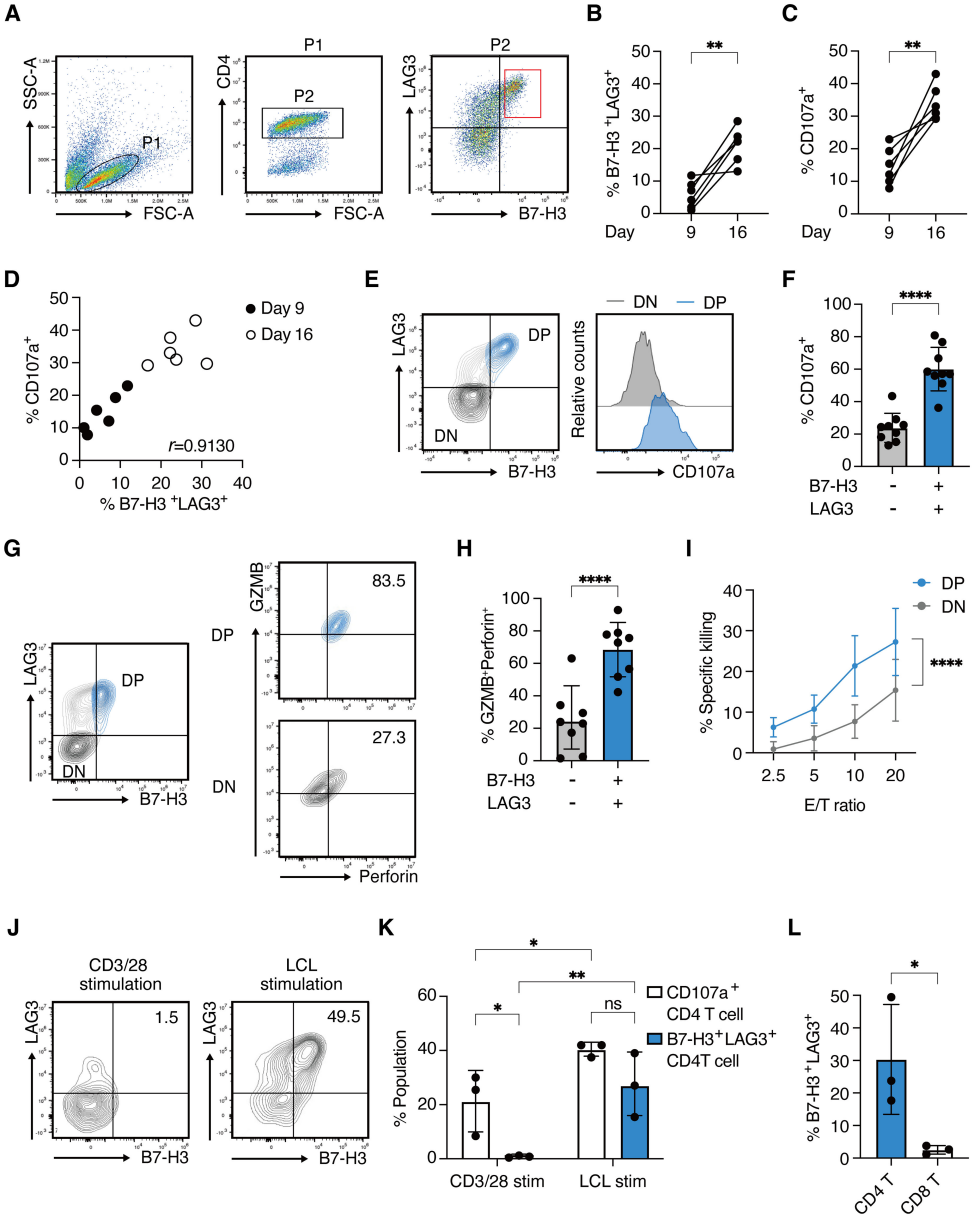
B7-H3 and LAG3 co-expression correlates with functional CD4 CTLs **(A)** Representative FACS plots for B7-H3 and LAG3 expression on CD4^+^ T cells on day 9 after stimulation with autologous LCLs. **(B, C)** Percentages of B7-H3 ^+^LAG3^+^ CD4^+^ T cells **(B)** and CD107a^+^ CD4^+^ T cells **(C)** on day 9 and day 16. Each line represents independent experiments from 5 healthy donors. **(D)** Correlation plot showing the percentages of CD107a^+^ CD4^+^ T cells and B7-H3^+^LAG3^+^ CD4^+^ T cells on day 9 and day 16. **(E)** Representative contour plot showing B7-H3 and LAG3 expression on CD4^+^ T cells (*left panel*) and histograms depicting CD107a expression in B7-H3/^+^LAG3^+^ (*blue*) and B7-H3^-^ LAG3^-^ (*light gray*) compartment (*right panel*) on day 16. **(F)** Percentage of CD107a^+^ population in the indicated compartments from 8 independent experiments from 5 healthy donors (n=8). **(G)** Representative contour plots of GZMB and Perforin expression in the B7-H3^+^LAG3^+^ (*right upper panel*) and B7-H3^-^LAG3^-^ (*right lower panel*) compartments gated on CD4^+^ T cells (*left panel*). **(H)** Percentage of GZMB^+^Perforin^+^ population (from **Figure 3G**) in the indicated compartments (n=8 from 5 healthy donors). **(I)** % specific killing on day 26-30 at the indicated E/T ratio of sorted B7-H3^+^LAG3^+^ CD4^+^ T cells (*blue*) and B7-H3^-^LAG3^-^CD4^+^ T cells (*light gray*).(4 independent assays from 3 donors.) **(J)** Representative contour plots of B7-H3 and LAG3 on CD4^+^ T cells 48 hours after stimulation with CD3/28 antibodies (*left panel*) or re-stimulation with autologous LCLs (*right panel*). **(K)** Percentages of CD107a^+^ population (*open bars*) and B7-H3^+^LAG3^+^ population (*blue filled bars*) are determined in indicated T cells from **Figure 3J** (n=3). **(L)** Percentages of B7-H3^+^LAG3^+^ population in indicated T cell fractions at 48 hours after re-stimulation with autologous LCLs (n=3). Data are mean ± SD with statistical significance determined by paired t-test **(B, C)**, unpaired t-test **(F, H, L)**, two-way ANOVA **(I)**, or one-way ANOVA **(K)**. The *p*-values are represented as *p < 0.05; **p < 0.01; ***p < 0.001; ****p < 0.0001. Pearson’s correlation analysis was used to calculate correlation coefficients **(D)**.

To examine the expression of B7-H3 (the protein encoded by *CD276*) and LAG3 on LCL-induced CD4 CTLs, CD4^+^ T cells from five healthy donors were stimulated with autologous LCLs on days 0, 7, and 14 and analyzed on days 9 and 16. On day 9, B7-H3 and LAG3-double positive (DP) cells were detected at low levels in the CD4-gated population, however, the frequency of these cells was significantly increased by repeated stimulation (**Figures 3A, B**). CD107a/LAMP-1 has been widely used as a marker for degranulating cytotoxic lymphocytes because surface expression is induced upon degranulation (24). Similar to B7-H3 and LAG3, the expression of CD107a increased in CD4^+^ T cells by restimulation (**Figure 3C**). The percentage of DP CD4^+^ T cells was strongly correlated with CD107a expression, indicating an association between B7-H3 and LAG3 expression and CD4^+^ T cell activation (**Figure 3D**). Consistently, B7-H3 and LAG3 on CD4^+^ T cells were upregulated early after stimulation with LCLs, peaking at 48 hours and downregulated after 72 hours, correlating with the kinetics of CD107a expression (**Supplementary Figure 3A**). DP cells exhibited higher CD107a expression than B7-H3 and LAG3-double negative (DN) cells (**Figures 3E, F**). Cytotoxic granules, GZMB and Perforin, were highly enriched in DP CD4^+^ T cells, alongside augmented T-bet expression, highlighting enhanced CTL differentiation and degranulation capacity in this subset (**Figures 3G, H**, and **Supplementary Figures 3B-D**). Additionally, DP cells showed higher expression of FASL, TRAIL, and IFN-γ, known as other killing pathways of CD4 CTLs, compared to DN cells (**Supplementary Figures 3E-G**). qRT-PCR analysis of sorted DP CD4^+^ T cells and DN CD4^+^ T cells revealed the expression levels of *CD276*, *LAG3*, *ZEB2*, *IFNg* were significantly higher in DP cells compared to DN cells. *RUNX3* and *TBX21* also showed a trend toward higher expression in DP cells (**Supplementary Figures 2H-M**). Indeed, DP cells exhibited enhanced target-killing activity compared to DN cells (**Figure 3I**).

Notably, B7-H3 and LAG3 were minimally upregulated by CD3/CD28 antibody stimulation, unlike CD107a (**Figures 3J, K**). Furthermore, B7-H3^+^LAG3^+^ CD8^+^ T cells were scarcely detected upon LCL stimulation, in contrast to the CD4^+^ T cells (**Figures 3L**). These results indicate that the co-expression of B7-H3 and LAG3 cells represents a unique feature of CD4 CTLs, characterized by target-dependent enhanced cytotoxic potential, and distinct from surface markers previously identified in CD8 CTLs.

### 3.4 B7-H3^+^LAG3^+^ CD4^+^T cells with enhanced cytotoxic capacity expand *ex vivo* from the bone marrow of pediatric B-ALL patients

**Figure 4:**
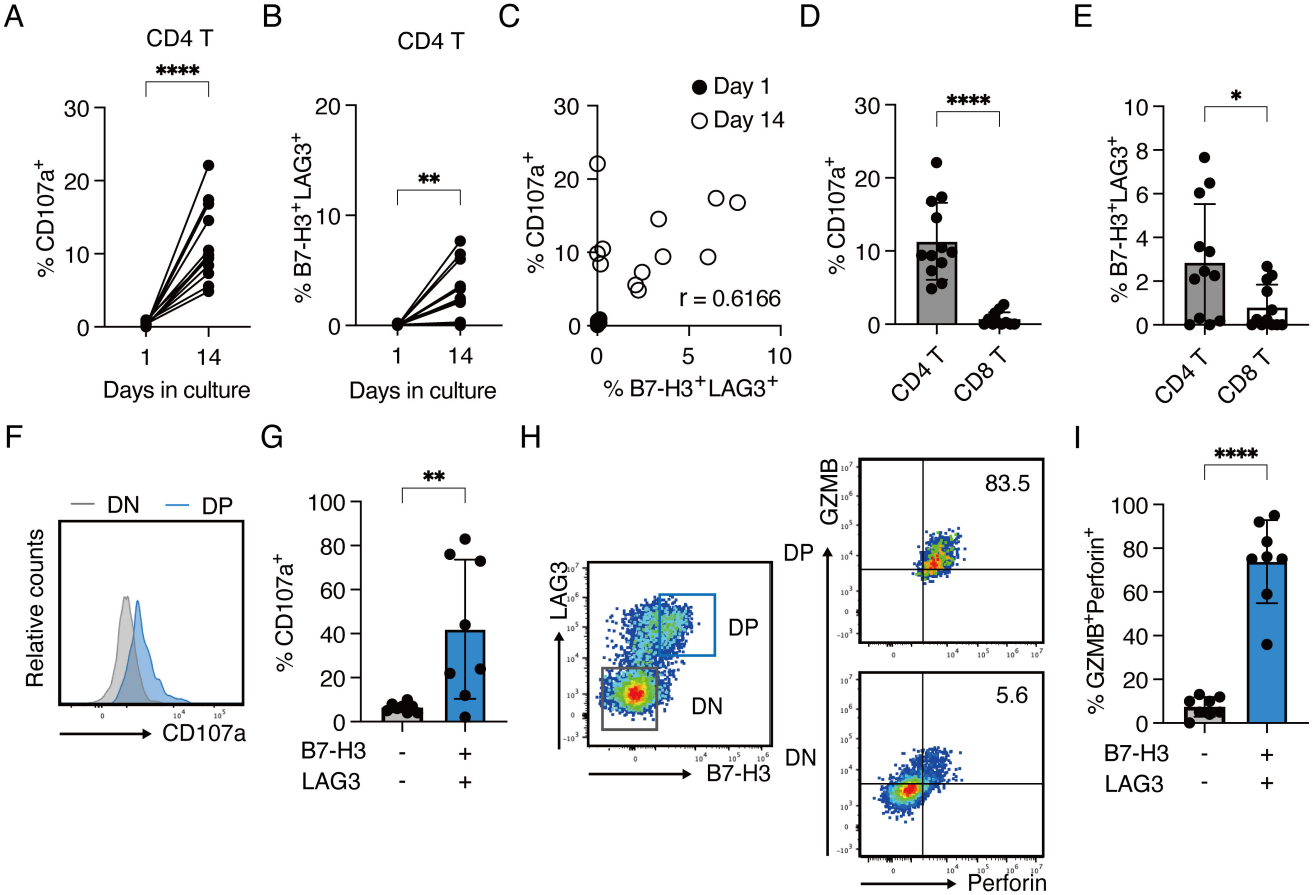
B7-H3^+^LAG3^+^ CD4^+^ T cells with enhanced cytotoxic capacity expand *ex vivo* from the bone marrow of pediatric B-ALL patients **(A–G)** Bone marrow mononuclear cells from 12 patients were cultured *ex vivo* with IL-2 and IL-7. Cells were analyzed by flow cytometry on day 1 and 14. Each line or dot represents an individual patient. **(A)** Percentages of CD107a-expressing cells in CD4^+^ T cells on day 1 and day 14 **(B)** Percentages of B7-H3^+^LAG3^+^ cells in CD4^+^ T cells on day 1 and day 14. **(C)** Correlation plot showing the percentages of CD107a^+^ CD4^+^ T cells and B7-H3^+^LAG3^+^ CD4^+^ T cells on day 1 and day 14. **(D)** Percentage of CD107a^+^ population in CD4^+^ and CD8^+^ T cells on day 14 (n=12) **(E)** Percentage of B7-H3^+^LAG3^+^ population in CD4^+^ and CD8^+^ T cells on day 14 (n=12) **(F)** Representative histograms for CD107a expression in B7-H3^+^LAG3^+^ (blue) and B7-H3-LAG3- (light gray) compartments on day 14. **(G)** Percentage of CD107a^+^ population in the indicated compartments. Samples with B7-H3^+^LAG3^+^ population at day 14 (from panel **B**) were analyzed (n=8). **(H)** Representative contour plots of GZMB and Perforin expression in the B7-H3/CD276^+^LAG3^+^ (right upper panel) and B7-H3/CD276^-^LAG3^-^ (right lower panel) compartments gated on CD4^+^ T cells (left panel). **(I)** Percentage of GZMB^+^Perforin^+^population (from panel **H**) in the indicated compartments. Samples with B7-H3^+^LAG3^+^ population at day 14 (from panel **B**) were analyzed (n=8). Data are mean ± SD with statistical significance determined by paired t-tests **(A, B)** and unpaired t-tests **(D, E, G, I)**. The p-values are represented as *p < 0.05; **p < 0.01; ***p < 0.001; ****p < 0.0001. Pearson’s correlation analysis was used to calculate correlation coefficients **(C)**.

Finally, to assess B7-H3 and LAG3 expression on CD4 CTLs in human tumor tissues, we analyzed bone marrow aspirates from twelve pediatric patients diagnosed with B-cell acute lymphoblastic leukemia (B-ALL). Given the high levels of human leukocyte antigen (HLA) class II expression on B-ALL cells, they could be potential targets for CD4 CTLs. When bone marrow mononuclear cells from the patients were harvested and cultured respectively *ex vivo*, the proportions of CD107a^+^ CD4^+^ T cells and B7-H3^+^/LAG3^+^ (DP) CD4^+^ T cells significantly increased after 14 days compared to the first day of culture (**Figures 4A, B**), with a mild correlation observed between the two populations (**Figure 4C**). In contrast, the proportions of CD107a^+^ CD8^+^ T cells and DP^+^ CD8^+^ T cells remained minimal, suggesting preferential activation of CD4^+^ T cells in response to B-ALL cells (**Figures 4D, E**). Notably, the DP CD4^+^ T cell population exhibited significantly higher CD107a expression than the B7-H3^-^/LAG3^-^ (DN) population, indicating enhanced cytotoxic degranulation capacity (**Figures 4F, G**). Furthermore, DP CD4^+^ T cells showed strong concordance with the expression of cytotoxic granules GZMB and Perforin (**Figures 4H, I**). These results underscore the co-expression of B7-H3 and LAG3 as potential indicators for identifying functional CD4 CTLs in patient-derived tissue samples.

## Discussion

In this study, we found that the co-expression of B7-H3 and LAG3 on activated CD4^+^ T cells strongly correlates with higher levels of cytotoxicity-related molecules such as GZMB, Perforin, and CD107a in both LCL-induced CD4 CTLs from human peripheral blood and CD4^+^ T cells derived from the bone marrow of B-ALL patients. Furthermore, we identified B7-H3^+^LAG3^+^ CD4^+^ T cells as a cytotoxic T cell population with multiple killing pathways such as GZMB/Perforin, FASL, TRAIL, and IFN-γ. These results suggest that B7-H3 and LAG3 co-expression may help identify CD4 CTLs with enhanced cytotoxic potential in human tumors.

Single-cell transcriptome analysis of tumor-infiltrating lymphocytes has revealed a heterogeneous population of CD4^+^ T cells with cytotoxic phenotypes varying across disease contexts (25–28), suggesting that the induction of CD4 CTLs depends on the immunological context and stimuli. While several markers of CD4 CTLs have been reported in mice and humans (12, 22, 29–32), our study took a novel approach by comparing the gene expression profiles of short-term functional CD4 CTLs and long-term dysfunctional CD4 CTLs. This comparison identified B7-H3 and LAG3 as cell surface proteins upregulated on highly cytotoxic CD4 CTLs, distinct from the markers shared with CD8 CTLs such as NKG7, CD29, SLAMF7, and NKG2D (20, 22, 27, 30, 33). Of note, co-expression of B7-H3 and LAG3 was predominantly observed in CD4 CTLs, suggesting their unique roles and distinct regulatory mechanisms in CD4^+^ T cells. Interestingly, B7-H3 and LAG3 expression was minimal in CD4 CTLs stimulated with anti-CD3/CD28, indicating that their induction requires interactions with target cells beyond TCR and CD28 signaling. This highlights a distinct regulatory mechanism compared to markers like CD107a. Future studies should clarify the molecular pathways governing B7-H3 and LAG3 expression in CD4 CTLs.

Given that B7-H3 and LAG3 are generally recognized as inhibitory molecules, their roles in functional CD4 CTLs are particularly intriguing. LAG3 is typically associated with Tregs and immune tolerance, preventing immune-mediated inflammation such as graft versus host disease (34). It is also known as a T cell exhaustion marker in addition to PD-1 and CTLA-4 (35). However, LAG3 expression has also been reported in autoimmune-related CD4^+^ T cells and inflammation-inducing CD4^+^ T cell subsets, suggesting diverse functions depending on the context (36, 37) B7-H3, a member of the B7 family, is known as an immune checkpoint protein that is highly expressed on cancer cells, often correlating with poor prognosis (38–41). In our study, B7-H3 and LAG3 co-expression enriched the CD4^+^ T cell population with higher cytotoxicity. One speculation is that highly activated CD4 CTLs may express inhibitory molecules such as B7-H3 and LAG3 to regulate their own excessive activation. However, the role of B7-H3 in T cells remains controversial, with some reports suggesting a co-stimulatory function in CD4^+^ T cell proliferation and IFN-γ production, whereas most studies indicate an inhibitory effect on T cell activity. This discrepancy may be attributed to the interaction of B7-H3 with multiple receptors or ligands, depending on the cell type or the microenvironment. Potential receptors, such as Triggering Receptor Expressed on Myeloid Cells (TREM)-like transcript 2 (TLT-2) and IL20RA, have been proposed, but their interactions with human B7-H3 have yet to be validated (42, 43). While cancer therapies targeting B7-H3 on the surface of tumor cells have shown promise, the effects of such therapies on T cells still require further investigation (44–46).

The induction of B7-H3^+^LAG3^+^ CD4^+^ T cells from the bone marrow of pediatric B-ALL patients and their correlation with high cytotoxic granule expression suggests a pivotal role of CD4^+^ T cells in controlling tumor growth in B-ALL, characterized by high HLA class II expression (47–49). Identifying B7-H3 and LAG3 as surface molecules expressed on CD4 CTLs with enhanced cytotoxic potential may provide new opportunities for manipulating CD4^+^ T cells in T cell therapies for B-ALL and other HLA class II-expressing tumors. The prolonged proliferation and sustained cytotoxic activity of CD4 CTLs compared to CD8 CTLs, as demonstrated in our study, suggests a unique advantage of CD4 CTLs in the immune response against tumors. This sustained cytotoxic activity may be crucial in maintaining long-term anti-tumor immunity, particularly in the tumor microenvironment where chronic antigen stimulation can lead to T cell exhaustion. Our study also highlights the importance of immunological context and stimuli in inducing CD4 CTLs. The distinct expression patterns of B7-H3 and LAG3 in response to specific antigenic stimulation suggest that these molecules could be useful for detecting tumor-reactive T cells, thereby, enhancing the efficacy of adoptive T-cell therapies. One limitation of this study is that it focused on MHC class II-positive tumors. Further research is required to explore the molecular mechanisms regulating B7-H3 and LAG3 expression on CD4 CTLs and to validate these findings in other tumor types.

In conclusion, the identification of B7-H3 and LAG3 co-expression as a characteristic feature of CD4 CTLs with enhanced cytotoxic potential may provide valuable insights into the role of CD4 CTLs in antitumor immunity. These molecules could serve as useful indicators for detecting and characterizing cytotoxic CD4^+^ T cells in human tissues, thereby facilitating future research and advancing therapeutic strategies that target CD4 CTLs in cancer and other diseases.

## Data Availability

The data that supported the findings of this study are openly available in NCBI with accession number PRJNA1148279. The materials used in this study are available upon the request, pending scientific review and a completed material transfer agreement.
