# Supplementary material for: Co-expression of B7-H3 and LAG3 represents cytotoxicity of CD4+ T cells in humans
Source: Front Immunol. 2025 Feb 25;16:1560383. doi: 10.3389/fimmu.2025.1560383 (PMC11893609; doi:10.3389/fimmu.2025.1560383)
Supplement: Supplementary file 1 [file DataSheet1.docx]

Supplementary Material

# Supplementary Figures and Tables

## Supplementary Figures

**Supplementary Figure 1. The expression of T-bet, Granzyme B, and Perforin in LCL.** (**A**-**C**) The bar graphs display the percentages of cells expressing T-bet (**A**), GZMB (**B**), and Perforin (**C**) in CD4 T cells from days 16-30 (4CTL-ST) [*orange*] and days 56-105 (4CTL-LT) [*purple*] from 4 healthy donors. Data are mean ± SD with statistical significance determined by paired t-test. The *p*-values are represented as **p* < 0.05; ***p* < 0.01; ****p* < 0.001; *****p* < 0.0001.

**Supplementary Figure 2.** **Normalized gene expression of the selected genes in LCL-stimulated or unstimulated CD4 T cells.** Total RNA was isolated from unstimulated CD4 T cells, 4CTL-ST, and 4CTL-LT. Relative gene expression levels of the indicated molecules were measured by quantitative real-time PCR and normalized to β2 microglobulin (n=2).

**Supplementary Figure 3.** **The expression of B7-H3/LAG3 and CD107a in LCL-stimulated CD4 T cells and cytotoxicity-related protein and gene expression in B7-H3^+^LAG3^+^ - and B7-H3^-^LAG3^-^ CD4 T cells**. (**A**-**G**) CD4 T cells were collected on day 21 after 4 rounds of restimulation with autologous LCLs and primed with LCLs at an effector-to-target ratio of 10:1. (**A**) Time course expression of CD107a (*open bars*) and co-expression of B7-H3/CD276 and LAG3 (*filled bars*) on CD4 T cells at the indicated time points. (**B**-**D**) Percentages of CD4 T cells expressing T-bet (**B**), GZMB (**C**), Perforin (**D**), FASL (**E**), TRAIL (**F**), and IFN-γ (**G**) in B7-H3/CD276^+^LAG3^+^ (*blue*) and B7-H3/CD276^-^LAG3^-^ (*light grey*) compartments. (**H-M**) CD4 T cells activated with 5 rounds of re-stimulation with LCLs were collected on day 30 and extracellularly stained with APC anti-B7-H3/CD276, PE/Cy7 anti-CD223, and PE- -anti-CD4. Total RNA was extracted from sorted B7-H3 ^+^LAG3^+^CD4 T cells and B7-H3 ^-^LAG3^-^CD4 T cells. Relative gene expression levels of *CD276* (**H**), *LAG3* (**I**), *ZEB2* (**J**), *IFN-γ* (**K**), *RUNX3* (**L)**, and *TBX21* (**M**) in B7-H3^+^LAG3^+^ (*blue*) and B7-H3^-^LAG3^-^ (*light grey*) compartments were measured by quantitative real-time PCR and normalized to β2 microglobulin (n=3). Data are mean ± SD with statistical significance determined by two-way ANOVA (**A**) and unpaired t-tests (**B**-**M**). The *p*-values are represented as **p* < 0.05; ***p*< 0.01; ****p* < 0.001; *****p* < 0.0001.

**Table S1. Serological characteristics of patients with B-ALL**

B-ALL, B cell acute lymphoblastic leukemia; CMV, cytomegalovirus; EBV, Epstein-Barr virus; VCA, viral capsid antigen; EA, early antigen; EBNA, EBV-associated nuclear antigen; NA, not assessed
